# Update on tick-borne rickettsioses in mainland Portugal: emerging threats and potential vectors

**DOI:** 10.1186/s13071-024-06627-5

**Published:** 2024-12-24

**Authors:** Leonardo Moerbeck, Ricardo Parreira, Gonçalo Seixas, Rita Velez, Ana Domingos, Sandra Antunes

**Affiliations:** 1 Global Health and Tropical Medicine- Institute of Hygiene and Tropical Medicine, NOVA University of Lisbon, Rua da Junqueira 100, 1349-008 Lisbon, Portugal; 2 Instituto de Higiene e Medicina Tropical, Universidade NOVA de Lisbon, Rua da Junqueira 100, 1349-008 Lisboa, Portugal; 3 Associate Lab in Translation and Innovation, Towards Global Health, LA-REAL, Rua da Junqueira 100, 1349-008 Lisbon, Portugal

**Keywords:** Tick-borne rickettsioses, TIBOLA, DEBONEL, SENLAT, MSF-like disease, Surveillance, One health

## Abstract

**Background:**

Tick-borne rickettsioses (TBR) are emerging, neglected, zoonoses, caused by intracellular α-proteobacteria of the genus* Rickettsia*, that pose a growing public health concern. The aim of the present study was to evaluate rickettsial infections in questing ticks collected from four different ecological areas in mainland Portugal.

**Methods:**

Over a two-year period, a total of 707 questing ticks were collected. Individual adult ticks and pooled nymphs were submitted to DNA extraction, followed by qPCR assays targeting the* gltA* rickettsial gene. Positive samples were then submitted to conventional PCR targeting the* gltA* and the* ompA* genes for phylogenetic analysis.

**Results:**

In total, eight tick species were identified:* Dermacentor marginatus*,* Haemaphysalis inermis*,* Haemaphysalis punctata*,* Hyalomma lusitanicum*,* Ixodes frontalis*,* Ixodes ricinus*,* Rhipicephalus pusillus*, and* Rhipicephalus sanguineus* sensu lato. Additionally, rickettsial infection was associated with seven of these species, with I. frontalis being the exception. Notably, the prevalence of Rickettsia spp. was 26.35%, with phylogenetic validation confirming infections with* R. helvetica*,* R. massiliae*,* R. monacensis*,* Candidatus* R. rioja, and* R. slovaca*.

**Conclusions:**

The present study highlights the necessity for ongoing surveillance to map and monitor both questing and feeding ticks, along with their vertebrate hosts. Effective control strategies are of utmost importance to mitigate the escalating threat of TBR. Additionally, the present study provides valuable epidemiological insights into TBR in Portugal, including the identification of* R. slovaca* infecting* I. ricinus* - an unconventional tick-pathogen relationship - and the first report of Candidatus R. rioja infecting D. marginatus in Portugal. In conclusion, this study contributes with valuable data regarding epidemiological results on ticks and TBR circulating in Portugal, emphasizing the importance of proactive measures to address this emerging public health challenge.

**Graphical abstract:**

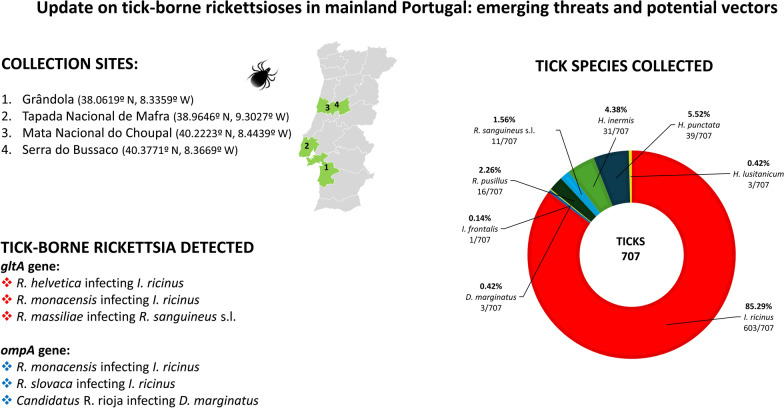

## Background

Tick-borne rickettsioses (TBR) are emerging, neglected zoonoses caused by obligate intracellular α-proteobacteria that belong to the spotted-fever group (SFG) of the genus *Rickettsia* [1]. Mediterranean Spotted Fever (MSF) is the most prevalent TBR in Europe, while tick-borne lymphadenopathy (TIBOLA) and Mediterranean spotted fever-like (MSF-like) diseases are the other TBRs in Europe that also pose significant threats to human health [2, 3]. MSF is caused by *Rickettsia conorii*, which has four subspecies, with both *R. conorii conorii* and *R. conorii israelensis* found in the Iberian Peninsula [1]. *Rhipicephalus sanguineus* sensu lato ticks not only are the primary vector but also act as reservoirs due to transstadial and transovarial transmission, with dogs potentially contributing to the persistence of MSF in the environment [1–3]. This TBR is characterized by fever, rash and the presence of an eschar of inoculation at the tick bite site. Most cases occur in summer, aligning with the peak activity of *R. sanguineus* s.l. [1–3]. TIBOLA is primarily caused by *Rickettsia slovaca*, *R. raoultii* and *Candidatus* R. rioja, while *Rickettsia helvetica*, *R. massiliae* and *R. monacensis* are known agents of MSF-like disease [3]. The first molecularly confirmed human case of TIBOLA, caused by *R. slovaca*, was reported in 1997 [4]. Since then, *R. raoultii* and *Candidatus* R. rioja have been not only associated with human cases of TIBOLA but have also been isolated from ticks removed from patients with this syndrome [5, 6]. *Dermacentor* ticks, specifically *D. marginatus* and *D. reticulatus*, are natural vectors of these rickettsial pathogens [2–4]. TIBOLA syndrome is characterized by the presence of neck lymphadenopathy, often linked to *Dermacentor* spp. bites on the human scalp. Other symptoms include fever, facial edema, alopecia around the tick bite site and, rarely, macular lesions on the limbs. These symptoms are the same regardless of the species of *Rickettsia* causing the infection [3]. Through the years, the knowledge about TIBOLA has expanded, and other terms such as “*Dermacentor*-borne necrosis erythema lymphadenopathy” (DEBONEL) and “scalp eschar and neck lymphadenopathy” (SENLAT) are also used to describe similar clinical manifestations or syndromes [2, 7].

MSF-like disease can be caused by different tick-borne rickettsiae within the SFG-rickettsiae, such as *R. helvetica*, *R. massiliae* and *R. monancensis* [3, 8]. Originally identified as the “Swiss agent” in 1979, and initially characterized as having unknown pathogenicity, *R. helvetica* was first isolated from *Ixodes ricinus* ticks in Switzerland [9]. Besides *I. ricinus*, *Ixodes ovatus*, *I. persulcatus* and *I. monospinus* are known vectors of this SFG-rickettsiae [10]. Additionally, *I. ricinus* is the natural reservoir of *R. helvetica*, as both transstadial and transovarial transmissions have been demonstrated in this tick species [10]. Until 1999, human pathogenicity was not confirmed; however, two fatal human cases were associated with this rickettsial species, in which Swedish patients showed perimyocarditis and vascular complications [11].

Another SFG rickettsia that causes MSF-like disease is *R. massiliae* [8]. It was initially isolated from *Rhipicephalus turanicus* and *R. sanguineus* s.l., both in France [12]. Later, it was confirmed that *R. turanicus* can transmit this rickettsial agent transovarially to its offspring [13]. Besides *Rhipicephalus turanicus*, *R. muhsamae*, *R. lunulatus* and *R. sulcatus* are additional known vectors of *R. massiliae* [10]. This pathogen was first isolated from humans in 1985 but was only identified in 2005, infecting an Italian patient displaying the symptom triad: fever, rash and necrotic eschar [14].

First isolated and identified in Germany infecting *I. ricinus* ticks, *R. monacensis* is one of several SFG-rickettsiae agents that cause MSF-like disease [15]. Although human cases are seldom reported, most symptoms are those typical of a rickettsiosis infection, including fever, headache and rash on the trunk and limbs [3]. Although *I. ricinus* has not yet been recognized as the natural vector of *R. monacensis*, previous studies have shown a high prevalence of this rickettsial agent infecting this tick species [16–18].

In Portugal, rickettsial agents responsible for TIBOLA and MSF-like disease have not only been reported in human cases, leading to hospitalization [19, 20], but have also been identified infecting their respective known vectors: *Dermacentor* spp. [21], *I. ricinus* [21, 22] and *R. turanicus* [23]. Additionally, putative vectors have been identified, with *R. massiliae* infecting *R. sanguineus* s.l. [24–26] and *R. monacensis* infecting *I. ricinus* [17, 27]. It is important to remember that ticks in southwestern Europe identified as *R. turanicus* based on morphological characteristics are genetically indistinguishable from *R. sanguineus* s.l., pointing to a high level of morphological polymorphism in this group [28–30]. Millán and colleagues [28] recently proposed and described *Rhipicephalus hibericus* as a new species in the *R. sanguineus* s.l. group, which would correspond to the tick species previously identified in this region as *R. turanicus*. Therefore, considering that the tick population and TBR are directly intertwined, effective campaigns concerning tick surveillance are of utmost importance for mapping and monitoring the distribution of these arthropods and the rickettsial agents they carry or can even transmit. Continuous surveillance not only helps in tracking threats to human and animal health but also in identifying new and emerging rickettsial agents and their tick association or putative vectors. In this context, the present study aims to evaluate rickettsial infections in questing ticks collected from four different ecological areas in mainland Portugal and provides the first molecular characterization of *R. slovaca* infecting *I. ricinus* tick species in mainland Portugal.

## Methods

### Study sites, tick sampling and morphological identification

**Fig. 1:**
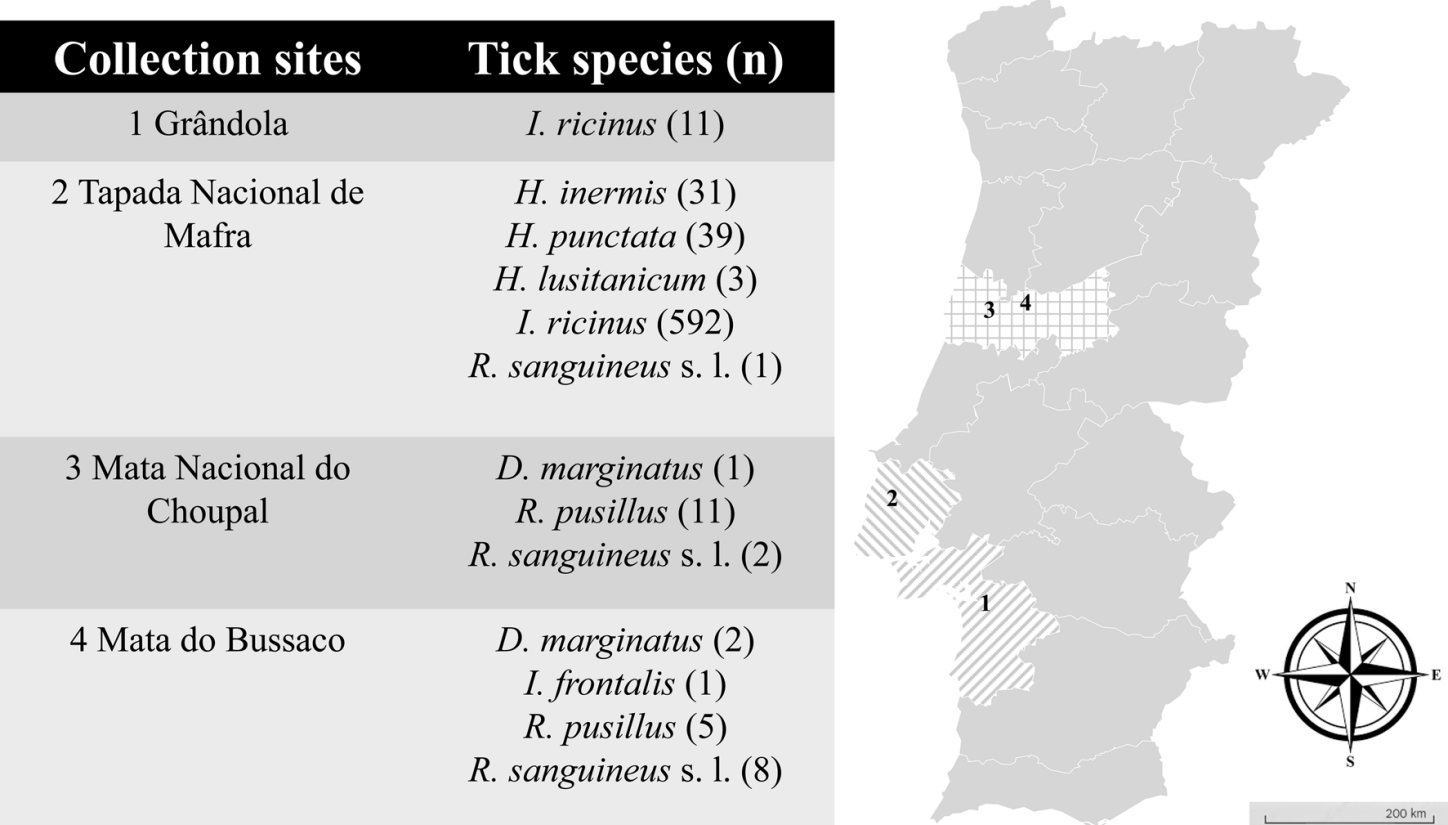
Geographical distribution of tick species in mainland Portugal from August 2018 until May 2021

Questing ticks were collected from four ecological areas in mainland Portugal from August 2018 until May 2021. The studied areas included Grândola (38º06′19.6"N, 8º33′59.7"W) with tick collections in February 2019, Mata Nacional do Choupal (40.2223ºN, 8.4439ºW) where sampling was conducted in June 2019, Mata do Bussaco (40.3771ºN, 8.3669ºW) where ticks were also collected in June 2019 and Tapada Nacional de Mafra (TNM) (38.9646ºN, 9.3027ºW) where tick collection occurred in December 2019 and May 2021 (Fig. 1), as previously described [31]. Each area was visited once, except TNM, which was visited twice. All ecological areas were selected based on previous reports that confirmed the presence of ticks and potential circulation of TBR [32]. During field campaigns, up to 20 tick specimens, collected by dragging or flagging methods, were deposited in a single 15-ml tube. To prevent tick dehydration, some green vegetation was placed inside the tube. All tick specimens were kept refrigerated in a box with ice packs until being brought to the laboratory for further analysis. Taxonomic classification of all specimens was carried out to species level based on morphological characters according to previously published taxonomic keys [33] using a Motic SMZ171 stereomicroscope.

### Tick nucleic acid extraction and detection of PCR inhibitors

Ticks were rinsed in pH 7.0 sterile phosphate-buffered saline solution (PBS) and pooled according to species, collection site, development stage and sex. All adult specimens were placed individually in a sterile 1.5-ml tube, while nymphs were pooled in groups of up to five specimens. Each sample was frozen in liquid nitrogen and crushed with sterile pestles. Genomic DNA (gDNA) was extracted using TRIzol^tm^ reagent (ThermoFisher Scientific, Carlsbad, CA), according to the manufacturer's protocol for isolation of nucleic acids from tissues. gDNA was resuspended in nuclease-free water, and concentrations were measured using a Qubit4 fluorometer (ThermoFisher Scientific, Carlsbad, CA). Afterwards, gDNA was stored at – 20 ºC until being used as a template for polymerase chain reaction (PCR) amplifications. Prior to any TBR screening, approximately 20% of gDNA samples were randomly selected to amplify the 18S*rDNA* tick gene fragment to confirm the absence of PCR inhibitors [34].

### Molecular detection of rickettsial DNA

Samples were screened targeting a 74-bp fragment of the *gltA* rickettsial gene by probe-based real-time PCR (qPCR). To amplify this rickettsial gene fragment, reactions were prepared in triplicate in 96-well plates using primers CS-F and CS-R with probe CS-P (Table 1), as previously described [35]. The positive control for qPCR reactions consisted of an eight-fold serial dilution of a synthesized fragment of the *Rickettsia rickettsii gltA* gene. This fragment, encompassing a 74-bp sequence (GenBank accession no. U59729), was synthesized as a gBlocks® gene fragment by Integrated DNA Technologies (IDT, Leuven, Belgium). Samples were considered positive in these qPCR screening assays if at least two or more of their replicates yielded the expected amplification result. To confirm positive results obtained from the qPCR screening assays and for phylogenetic purposes, samples were subsequently tested by conventional PCR for both the outer membrane protein A gene (*ompA*) and *gltA* gene [36] Table 1.

**Table 1:** Primers used for amplification of rickettsial gene fragments

Target gene	Primer (nucleotide sequence 5′-3′)	Method	Amplicon size (bp)	Reference
*gltA*	CS-F: TCG CAA ATG TTC ACG GTA CTT T	qPCR	74	[35]
	CS-R: TCG TGC ATT TCT TTC CAT TGTG			
	CS-P (probe) 6-FAM-TGC AAT AGC AAG AAC CGT AGG CTG GAT G-BHQ-1			
	RpCS.877p: GGGGGCCTGCTCACGGCGG	PCR	381	[36]
	RpCS.1258n: ATTGCAAAAAGTACAGTGAACA			
*ompA*	Rrl9O.70p: ATGGCGAATATTTCTCCAAAA	PCR	532	[36]
	Rrl9O.602n: AGTGCAGCATTCGCTCCCCCT			

### Statistical analysis

The standard prevalence of adult ticks was calculated based on individually tested samples. The prevalence of rickettsial infection in nymphs was estimated using the minimum infection rate (MIR) [37] and adjusted for pools of varying sizes and individual samples. MIR was calculated by dividing the number of positive pools, confirmed by qPCR when two or more replicates produced the expected amplification, by the total number of ticks tested. The overall prevalence of infection, combining adult ticks (tested individually) and nymphs (tested in pools using the MIR), was determined by weighting the prevalence of each group according to the number of ticks tested in each category. Here, the total number of ticks tested was calculated by summing the number of ticks across all pools and individual samples to account for different pool sizes and individual samples.

### DNA sequencing and phylogenetic analysis

All the *gltA* and *ompA* amplicons, obtained by conventional PCR, were purified using the NZyGelpure kit (NZYtech, Lisbon, Portugal) according to the user guide protocol. Afterwards, they were sent to StabVida (Caparica, Portugal) for Sanger sequencing. All obtained sequences were aligned and compared to those already deposited at the NCBI (National Center for Biotechnology Information) nucleotide database (https://blast.ncbi.nlm.nih.gov/). Obtained sequences corresponding to the citrate synthase gene (*gltA*) were deposited at GenBank under accession numbers PP935122–PP935137, while those corresponding to the outer membrane protein A gene (*ompA*) were assigned accession numbers PP935138-PP935154.

Datasets were created with short reads obtained as a result of Sanger sequencing, reference sequences previously deposited in GenBank and sequences returned from Megablast research [Nucleotide BLAST: Search nucleotide databases using a nucleotide query (nih.gov)], which demonstrated the best “query cover” and “identity percentage” rates, always from different studies. All sequences in each gene-specific dataset were aligned using MAFFT (https://mafft.cbrc.jp/alignment/server/), and the obtained multiple sequence alignments were edited GBlocks 0.91b (http://phylogeny.lirmm.fr/phylo_cgi/one_task.cgi?task_type=gblocks). Two different approaches were used to construct the phylogenetic trees to minimize the biases that may be introduced by the methods used. At first, phylogenetic trees were constructed based on neighbor-joining (NJ) analysis with genetic distance matrixes corrected with Kimura two-parameter (K2P) substitution model using MEGA v.10 [38]. Afterwards, another set of phylogenetic trees was constructed based on the maximum-likelihood (ML) optimization criterion, using the best-fit model for each sequence dataset, according to BIC (Bayesian Information Criterion), as defined by IQ-TREE web server model selection tool (http://iqtree.cibiv.univie.ac.at/). In both cases, the topological soundness of the obtained trees was assessed by bootstrapping with 1000 resampling of the original sequence data. The graphic representation of phylogenetic trees selected for the current study was that from the NJ analysis once the results of both phylogenetic analysis approaches (NJ and ML) demonstrated identical topological characteristics, i.e. the same nodes, branches and bootstrap values.

## Results

A total of 707 questing ticks (538 nymphs, 76 males, 93 females) were gathered during field collection, attaining a total of eight species: *D. marginatus* (*n* = 3; 0.42%), *Haemaphysalis inermis* (*n* = 31; 4.38%), *Haemaphysalis punctata* (*n* = 39; 5.52%), *Hyalomma lusitanicum* (*n* = 3; 0.42%), *Ixodes frontalis* (*n* = 1; 0.15%), *I. ricinus* (*n* = 603; 85.29%), *Rhipicephalus pusillus* (*n* = 16; 2.26%) and *R. sanguineus* s.l. (*n* = 11; 1.56%). Among the *I. ricinus* specimens collected, there were 86 adults (*n* = 52 males and *n* = 34 females) and 538 nymphs (Table 2).

**Table 2:** Prevalence and phylogenetic confirmation of tick-borne rickettsiae in collected tick species across different ecological areas

Region/tick species	Stage	qPCR samples *glt*A (+/screened/adult prevalence^b^/MIR^a^)	*Rickettsia spp.* sequenced samples	
Grândola			*glt*A phylogenetic confirmation (sample accession number)	*omp*A phylogenetic confirmation (sample accession number)
*Ixodes ricinus*	9 M, 2F	11/11/100%^b^	*R. helvetica* (PP935134) *R. monacensis* (PP935122, PP935123, PP935124, PP935125, PP935126, PP935127, PP935128)	*R. monacensis* (PP935138, PP935139, PP935140, PP935141, PP935142, PP935143)
Mata do Bussaco				
*Dermacentor marginatus*	2F	2/2/100%^b^		*Candidatus* R. rioja (PP935154)
*Ixodes frontalis*	1N	0/1/0%^b^		
*Rhipicephalus pusillus*	5F	2/5/40%^b^		
*Rhipicephalus sanguineus* s.l	1 M, 7F	8/8/100%^b^	*R. massiliae* (PP935137)	
Mata do Choupal				
*Dermacentor marginatus*	1 M	1/1/100%^b^		
*Rhipicephalus pusillus*	5 M, 6F	2/11/18.2%^b^		
*Rhipicephalus sanguineus* s.l	2F	1/2/50%^b^	*R. massiliae* (PP935136)	
Tapada Nacional de Mafra				
*Haemaphysalis inermis*	11 M, 20F	4/31/12.9%^b^		
*Haemaphysalis punctata*	5 M, 14F, 20N	2/19/13%^b^ 1/4/5%^a^		
*Hyalomma lusitanicum*	3F	1/3/33.3%^b^		
*Ixodes ricinus*	43 M, 32F, 517N	13/75/17.3%^b^ 24/103/4.6%^a^	*R. helvetica* (PP935135) *R. monacensis* (PP935129, PP935130, PP935131, PP935132, PP935133)	*R. monancensis* (PP935144) *R. monancesis* (PP935145, PP935146, PP935147, PP935148, PP935149, PP935150, PP935151, PP935152) *R. slovaca* (PP935153)
* Rhipicephalus sanguineus* s.l	1 M	1/1/100%^b^		
Total	538N; 76 M; 93F	73/277/10.31%^c^		

The minimum infection rate^a^ (MIR) was calculated when TBR-infected samples were detected in nymph pools, and standard prevalence^b^ infection was tested in adult ticks (individual specimen). Samples that did not reach phylogenetic resolution to species level were considered positive for *Rickettsia* spp. infection for the purposes of calculating standard prevalence^b^, MIR^a^ and the overall prevalence^**c**^, which was determined by weighting the prevalence of each group according to the number of ticks tested in each category (adult ticks—tested individually and nymphs—tested in pools using the MIR)

The overall prevalence of rickettsial infection in the 277 samples where the presence of rickettsial DNA was investigated by qPCR assays (for detection of the *gltA* gene) was 10.31%. Among nymphs, the prevalence determined by MIR was 4.64%, while in individually tested adult samples, the obtained prevalence was 28.4% (Table 2). Moreover, all tick species, except for *I. frontalis* collected from Mata do Bussaco, yielded positive amplification results.

**Fig. 2:**
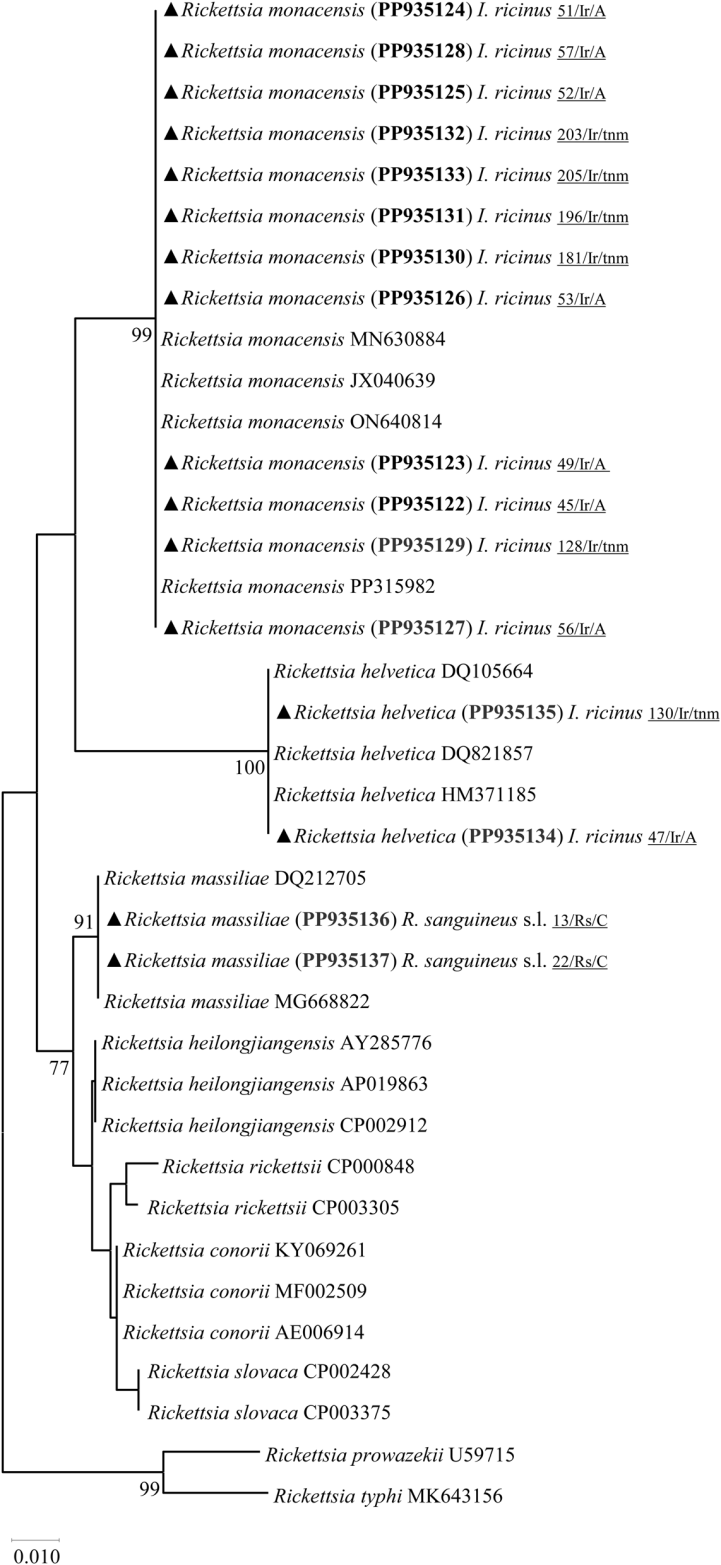
Phylogenetic tree constructed and displayed by the neighbor-joining method with Kimura’s two-parameter evolution model from partial sequences of the *gltA* gene. The same topographic representation was obtained by the maximum likelihood method with transition-intermediary model (TIM) + F evolution model from partial sequences of the *gltA* gene according to the BIC (Bayesian information criterion), as defined by IQ-TREE web server model selection. Bootstrap values were obtained from 1000 replications and are indicated at the nodes of the respective branches (only values ≥ 75%). All *Rickettsia* spp. sequences obtained during this work are highlighted with a triangle and its respective accession, both in bold format, and clone names are underlined

Phylogenetic analysis based on the citrate synthase (*gltA*) gene revealed that *R. helvetica* infected one male (PP935134) and one pool of nymphs (PP935135) from *I. ricinus* ticks. On the other hand, *R. massiliae* was found in association with two *R. sanguineus* s.l. female specimens (PP935136, PP935137), while *R. monacensis* was found exclusively in *I. ricinus* ticks. This included six males (PP935122, PP935123, PP935124, PP935125, PP935126, PP935128), one female (PP935127) and five pools of nymphs (PP935129, PP935130, PP935131, PP935132, PP935133) (Table 2) (Fig. 2).

**Fig. 3:**
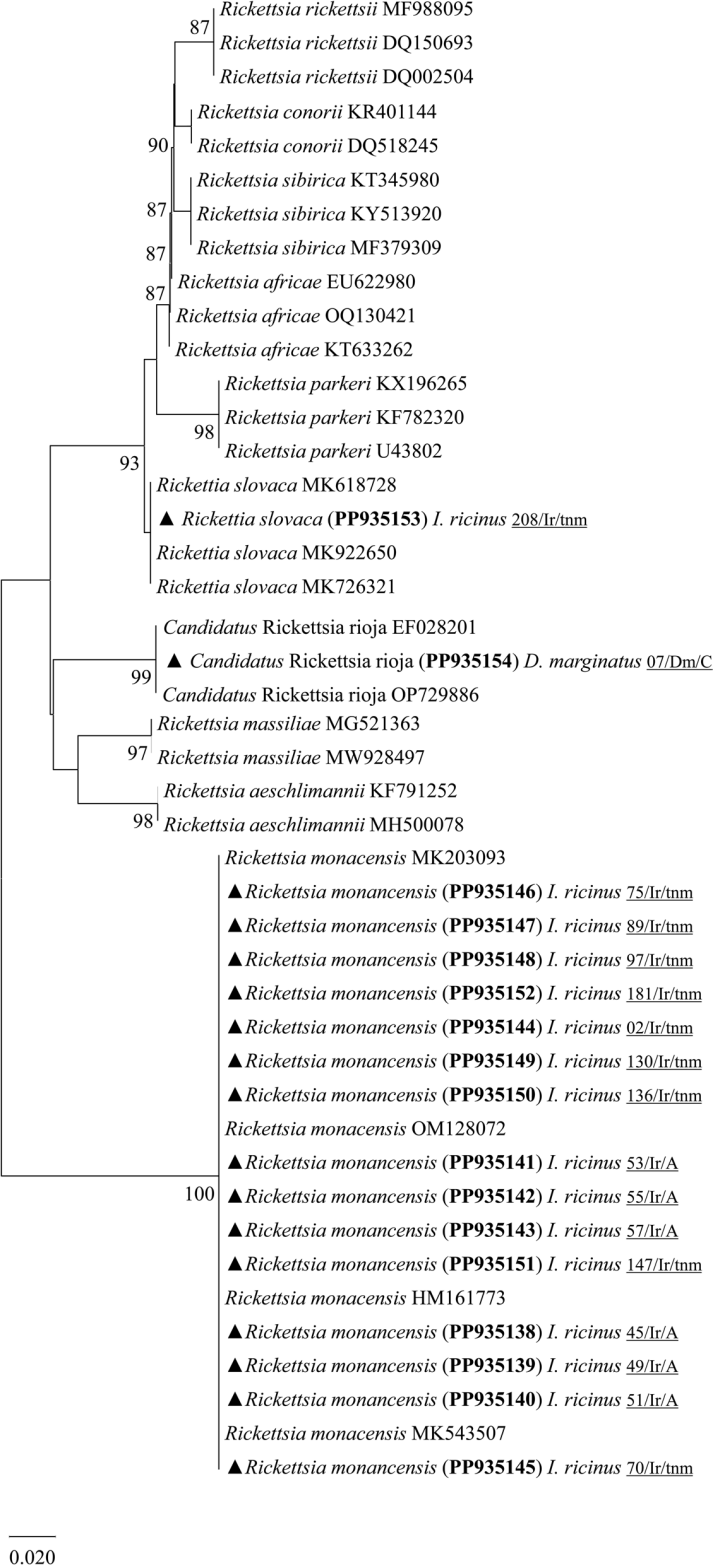
Phylogenetic tree constructed and displayed by the neighbor-joining method with Kimura’s two-parameter evolution model from partial sequences of the *ompA* gene. The same topographic representation was obtained by the maximum likelihood method with Tamura-Nei (TNe) evolution model from partial sequences of the *ompA* gene according to the BIC (Bayesian information criterion), as defined by IQ-TREE web server model selection. Bootstrap values were obtained from 1000 replications and are indicated at the nodes of the respective branches (only values ≥ 75%). All *Rickettsia* spp. sequences obtained during this work are highlighted with a triangle and its respective accession, both in bold format, and clone names are underlined

Regarding the phylogenetic analysis based on the outer membrane protein A (*ompA*) gene, the findings were as follows: *Candidatus* R. rioja infecting one female of *D. marginatus* (PP935154); *R. slovaca* infecting one pool of *I. ricinus* nymphs (PP935153); *R. monacensis* was detected exclusively in *I. ricinus* ticks, including one female (PP935144), six males (PP935138, PP935139, PP935140, PP935141, PP935142, PP935143) and eight pools of nymphs (PP935145, PP935146, PP935147, PP935148, PP935149, PP935150, PP935151, PP935152) (Table 2) (Fig. 3).

## Discussion

The present study provides molecular evidence for the circulation of several tick-borne rickettsiae, including, *R. helvetica*, *R. massiliae*, *R. monacensis*, *Candidatus* R. rioja and *R. slovaca*, in Ixodidae questing ticks collected from the field in mainland Portugal. It is important to emphasize that the overall prevalence of 10.31% was calculated using the minimum infection rate (MIR) for pooled tick samples (composed exclusively of nymphs), a conservative approach that assumes that there is only one infected tick per positive pool, even if multiple ticks may actually be infected. In contrast, the prevalence for adult ticks, which were individually analyzed, was determined through standard methods. Therefore, the estimated prevalence of *Rickettsia* spp. in ticks may be underestimated, and this value should be regarded as a lower bound for the actual infection rate [37]. Notably, the qPCR assay used in this study, targeting the gltA gene, was a probe-based method recognized for its high specificity and sensitivity [35]. This enhances confidence in the accuracy of the qPCR-positive results, even in cases where conventional PCR assays failed to amplify larger fragments of the *gltA* or *ompA* genes [35]. The inability of conventional PCR to confirm some qPCR-positive results can likely be attributed to its lower sensitivity, particularly in samples with degraded DNA or low target abundance. To ensure a comprehensive representation of *Rickettsia* spp.’s prevalence, all qPCR-positive samples were included in the calculations. This approach leverages the high sensitivity and specificity of the probe-based qPCR assay to provide a reliable estimation of Rickettsia spp. prevalence, while acknowledging the limitations of conventional PCR and the potential for minor overestimation in rare cases of nonspecific amplification [39]. The present report discloses the infection of *I. ricinus* with *R. helvetica*, one of the SFG-rickettsiae that causes MSF-like disease, via the association of species-specific *gltA* sequences obtained in the course of this work (PP935134-PP935135) to a topologically stable monophyletic group in a gene-specific phylogenetic tree (Fig. 2). Although there are no records of human MSF-like disease caused by *R. helvetica* in Portugal, previous reports have shown its presence in its known natural vector and reservoir, *I. ricinus* [22, 40]. Moreover, *R. helvetica* has also been detected infecting lizards (*Teira dugesii*) and *I. ricinus* ticks removed from the latter, suggesting that this vertebrate might act as a potential reservoir, maintaining *R. helvetica* throughout the enzootic cycle [22]. Additionally, *Ixodes ventalloi* has also been reported to be infected with *R. helvetica* in Portugal [41] although the competence of *I. ventalloi* as a vector for this bacterium remains unknown [42].

Two *gltA* gene sequences (PP935136, PP935137), obtained from infected *R. sanguineus* s.l. females, showed infection by *R. massiliae* forming a well-supported clade in the phylogenetic tree, indicating its clear genetic distinction within the SFG rickettsiae, another causative agent of MSF-like disease (Fig. 2). These outcomes align with previous studies conducted in Portugal [17, 24–26]. Additionally, *R. massiliae* has been reported infecting *R. turanicus* [41], a tick species recognized as both a vector and a reservoir for this SFG-rickettsiae [13]. While no human disease cases caused by *R. massiliae* have been reported in Portugal, infections have been documented in dogs (*Canis lupus familiaris*) [26], sheep (*Ovis aries*) [24] and ticks collected from these animals. Although not all infected dogs exhibited clinical signs, when they occurred, those most frequently observed were anorexia and jaundice. On the other hand, the most prevalent hematological abnormalities were moderate to mild thrombocytopenia, followed by monocytosis [26]. Conversely, none of the sheep exhibited relevant clinical signs in response to rickettsial infection [24]. As sheep are parasitized by the same tick species that accidentally parasitize humans [43], and since these domestic animals do not exhibit signs of a rickettsial infection, these intertwined scenarios not only elevate the transmission risk of *R. massiliae* to humans but also favor the circulation, as well as maintenance, of a possible interspecific domestic cycle of this pathogen.

DNA fragments of *R. monacensis* from both *gltA* or *ompA* were detected exclusively in *I. ricinus* ticks in a well-supported clade, confirming its status as a distinct species and common presence in these ticks (Figs. 2 and 3). These results align with previous studies in mainland Portugal and Madeira Island, which have shown not only the presence of this pathogen in questing *I. ricinus* [17, 27] but also in lizards (*T. dugesii*) and their parasitizing ticks [22]. Recently, the first confirmed human case of MSF-like disease caused by *R. monacensis* was reported in Portugal. The patient showed fever, rash, myalgia, fatigue and anorexia [19]. Moreover, an engorged female *I. ricinus* tick was removed from this patient, and both the tick and the eschar biopsy sample confirmed infection by this rickettsial agent [19].

Concerning the *ompA* rickettsial gene, the present study also confirms the presence of *R. slovaca* and *Candidatus* R. rioja in a pooled sample of *I. ricinus* nymphs and a *D. marginatus* female, respectively. Both, causative agents of TIBOLA reported here show that their phylogenetic placement is robust, confirming a clear genetic distinction from other rickettsial agents. Initially, *R. slovaca* was identified as the only causative agent of TIBOLA involving *D. marginatus* tick species [4]. Over the years, not only has *D. marginatus* been considered the natural vector of *R. slovaca*, but also *D. reticulatus* has been incriminated as a potential vector [2, 3]. Nevertheless, the present study reports *I. ricinus* infected with *R. slovaca* (PP935153), an uncommon but increasingly frequent finding, especially in areas where this SFG-rickettsiae has been reported infecting not only both questing and feeding ticks but also their respective vertebrate hosts [16, 44, 45]. Furthermore, the pooled sample, consisting of five *I. ricinus* nymphs, was collected at TNM, a forest-like region with rich fauna featuring large mammals, such as the red deer (*Cervus elaphus*), medium-sized mammals, like the European fallow deer (*Dama dama*), and smaller ones, such as foxes (*Vulpes vulpes*) and wild boar (*Sus scrofa*) (BNP-Tapada de Mafra. Available online: http://bibliografia.bnportugal.gov.pt/bnp/bnp.exe/registo?1687264). In this context, when different vertebrate hosts sympatrically coexist in such a rich environment, this complex and dynamic scenario not only facilitates the parasitism of different hosts by the same tick species but also contributes to the maintenance of the enzootic/sylvatic cycle of *Rickettsia* spp. within this region. Although scarce in Portugal, there have been at least three previously reported cases of human TIBOLA, all caused by *R. slovaca* [20].

The other causative agent of TIBOLA detected in this study was *Candidatus* R. rioja, infecting one female *D. marginatus* specimen. Although there are no records of humans, or ticks infected with this rickettsial agent in Portugal, its association with ticks has already been confirmed in Spain, where a patient exhibiting signs of TIBOLA syndrome had a *D. marginatus* tick attached to her scalp, where infection with *Candidatus* R. rioja was later confirmed [5]. Later, a biological sample from the same patient confirmed the first TIBOLA/DEBONEL/SENLAT human case caused by *Candidatus* R. rioja [6]. Most recently, once again in neighbouring Spain, *Candidatus* R. rioja was not only identified as the most frequent causative rickettsial agent of TIBOLA [46] but also, in two specific regions bordering Portugal, this SFG-rickettsiae was detected in both wild ungulates (*C. elaphus* and *S. scrofa*) and *D. marginatus* ticks parasitizing them [47]. The scenario presented in these regions on the opposite side of the Portuguese border raises some concern regarding their possible circulation within the Portuguese territory, despite the apparent low prevalence of *Rickettsia* sp. infection in wild animals, as these may support their maintenance in *D. marginatus*. Indeed, this scenario could potentially be extended to any of the regions within the Portuguese territory where *D. marginatus*, *C. elaphus* and *S. scrofa* have been found to coexist [47].

## Conclusions

The present study provides molecular characterization of several tick-borne rickettsiae circulating in Portugal, including *R. helvetica*, *R. massiliae*, *R. monacensis*, *Candidatus* R. rioja and *R. slovaca*, detected in naturally infected Ixodidae ticks. The findings regarding TBR that cause MSF-like disease, specifically, *R. helvetica*, *R. massiliae* and *R. monacensis*, confirm their presence in their respective vectors and reservoirs. Additionally, the detection in this work of *Candidatus* R. rioja and *R. slovaca*, two of the causative agents of TIBOLA, not only confirms their natural presence in association with their natural vector (*D. marginatus*) but also confirms the latter in an unusual interspecific relationship with an anthropophilic tick such as *I. ricinus*.

Overall, these outcomes highlight the need for a continued surveillance program and the implementation of effective prevention and control strategies to address the emerging threat of TBR in Portugal. Future studies should focus on the vector competence of tick species, particularly those associated with unusual TBR, as well as the ecological dynamics that influence the maintenance and transmission of rickettsial agents within enzootic or sylvatic cycles. Additionally, the collection of anthropophilic ticks is essential, as it provides valuable data for mapping and monitoring eco-epidemiological changes. This comprehensive data collection will enhance the development of predictive risk models, offering physicians updated information on the epidemiological situation and supporting the One Health approach.

To the best of our knowledge, this is the first report of *R. slovaca* infecting *I. ricinus* and the first detection of *Candidatus* R. rioja infecting *D. marginatus*, its natural vector, in Portugal.

## Data Availability

No datasets were generated or analysed during the current study.
